# D-cycloserine augmentation of behavior therapy for anxiety and obsessive-compulsive disorders: A meta-analysis

**DOI:** 10.1371/journal.pone.0173660

**Published:** 2017-03-10

**Authors:** Paul-Christian Bürkner, Nadine Bittner, Heinz Holling, Ulrike Buhlmann

**Affiliations:** Institute of Psychology, University of Münster, Münster, Germany; Chiba Daigaku, JAPAN

## Abstract

**Objective:**

The present meta-analysis investigates whether the antibiotic D-cycloserine (DCS), a partial agonist at the glutamatergic N-methyl-D-aspartate receptor, can augment the effect of behavior therapy in humans with anxiety and obsessive-compulsive disorders.

**Method:**

A keyword-based computer search was conducted using common electronic databases. Only studies investigating the effect of DCS in humans with anxiety and obsessive-compulsive disorders were included, resulting in 23 studies with a combined sample size of 1314 patients. Effect sizes were coded as Hedges’ g and SMCC, the latter also incorporating differences in pre-treatment values. Bayesian multilevel meta-analysis was applied to take dependencies of effect sizes obtained from the same study into account.

**Results:**

While previous meta-analyses found small to moderate improvements, the current results including the most recent research indicate that the overall effect of DCS is very small and almost indistinguishable from zero (*g* = -0.12, *CI* = [-0.27, 0.02]; *SMCC* = -0.10, *CI* = [-0.29, 0.07]). Slightly larger effects were found for social anxious patients. Further, study quality and year of publication were relevant moderators, with higher quality / more recent studies reported smaller effects of DCS.

**Conclusions:**

These findings raise the question of the usefulness of DCS as an augmentation of exposure therapy for anxiety and obsessive-compulsive disorders. At least, it seems to be less promising than initially thought. The fact that study quality was inversely related to the reported effect sizes underlines the importance of high quality primary research in order to avoid over-estimation of treatment effects in clinical psychology.

## Theoretical introduction

Anxiety disorders are among the most prevalent mental disorders worldwide [1]. In addition, obsessive-compulsive disorder (OCD), a former anxiety disorder according to the DSM-IV [2], is also quite common with an estimated lifetime prevalence of 2.3% [3]. It tends to be chronic, if left untreated, with a strong negative impact on psychosocial functioning and quality of life as well as a high mortality rate [4,5].

Cognitive behavioral therapy (CBT) including elements of exposure as well as psychotropic medication such as selective serotonin reuptake inhibitors (SSRIs) are considered the treatments of first choice for anxiety disorders and OCD [6,7]. Interestingly, the combined treatment of CBT and psychotropic medication does not seem to provide an additional benefit than CBT alone in a series of anxiety disorders such as panic disorder [8], social anxiety disorder [9], or OCD [10]. In addition, many patients still remain symptomatic after the completion of these treatments [11,12], further stressing the need to develop innovative treatment approaches for anxiety disorders and OCD.

There has been a growing body of research with respect to basic learning and memory processes involved in exposure therapy and their pharmacological enhancement [13]. Specifically, N-methyl-D-aspartate (NMDA) receptors located in the amygdala seem to play a significant role in the process of extinction of fear [14,15]. Consequently, the effects of NMDA receptor agonists such as the partial agonist D-cycloserine (DCS) on the enhancement of extinction have been examined in humans [15–17]. However, the exact mechanisms of why DCS might work as an augmentation of CBT are still unknown. There is some evidence that DCS facilitates the consolidation of new memories [18]. Moreover, there is evidence that the chronic use of NMDA receptor agonists desensitizes the glycine site of the NMDA receptor [19], which is consistent with clinical findings in the treatment of psychological disorders that the augmenting effects of DCS deteriorates when administered over an extended period of treatment sessions [20]. This raises the important question about the usefulness of DCS when administered over a more typical period of time used for CBT (e.g., 16 sessions or higher).

Several studies have already investigated the usefulness of DCS as an augmentation strategy for CBT, although the deteriorating effect of DCS has not specifically been the focus of the previous research so far. Ressler et al. [21], for example, examined the DCS augmentation of extinction learning in patients with acrophobia. Specifically, 28 patients were treated with two sessions of CBT including exposure. Prior to these sessions, single doses of DCS (or placebo) were administered. Patients in the DCS (vs. placebo) group improved significantly more with respect to their symptom severity. Further, this improvement was still detectable three months after the treatment was completed [21]. This first study was followed by several other studies on anxiety disorders including agoraphobia / panic disorder [22,23], OCD [20,24–29], post-traumatic stress disorder [30–33], social anxiety disorder [34–37], and specific phobia [21,38,39]. However, these findings were somewhat mixed with respect to the usefulness of DCS as a cognitive enhancer for behavior therapy. For example, early findings from Hofmann et al. [35] and Guastella et al. [34] indicated a strong effect of DCS (vs. placebo) as an augmentation strategy for a 5-session treatment of social anxiety disorder. Later findings, however, found no clear advantage of DCS at the end of treatment (i.e., 8 sessions or higher) but a faster treatment response earlier in treatment in psychological disorders such as social anxiety disorder [36], agoraphobia and panic disorder [23], or OCD [29]. Nonetheless, a faster symptom reduction in the beginning of treatment can have additional benefits, e.g., with respect to limited CBT therapist time and lower treatment costs [40].

As a consequence of these somewhat mixed findings, four meta-analyses were conducted thus far examining whether DCS is an effective enhancer of behavior therapy both in animal and human populations [15,41–43]. In the first meta-analysis by Norberg et al. [15], the effects of DCS for the facilitation of fear extinction in animals and behavior therapy in humans with anxiety disorders and OCD as well as the effects of dose, timing and number of DCS administration were examined. 15 primary studies providing 30 different samples were included in their analysis. Norberg et al. [15] found that DCS leads to an improvement of extinction learning in both animal and human samples with statistically detectable effects even at follow-up assessment. Further, they concluded that DCS is most efficient when administered immediately before or after exposure and in a limited number of sessions.

Bontempo et al. [41] included nine studies in their meta-analysis on DCS augmentation of behavior therapy for the treatment of anxiety disorders. With regard to potential moderating variables, the authors examined the influence of DCS timing before treatment, number of DCS doses administered and amount of dosage, type of anxiety disorder, number of received treatment sessions as well as the methodological quality of the included studies. In sum, this meta-analysis also supported the assumption that DCS significantly enhances CBT including EX for the treatment of anxiety disorders. But no effects could be found for any of the analyzed potentially moderating variables.

Rodrigues et al. [42] identified 13 studies with different types of anxiety disorders through electronic search in relevant databases. Their overall result was in line with the two former meta-analyses as the authors were also able to demonstrate that DCS has an enhancing effect on exposure-based treatment of anxiety disorders, although the effect was somewhat smaller than in the previous meta-analyses.

Parallel to our own meta-analysis (and unaware to us until publication in January 2017), Mataix-Cols et al. [43] conducted a comprehensive meta-analysis on raw data of 1047 patients showing a small overall effect of DCS at post-treatment and follow-up. In the light of recent discussions that the main benefit of DCS may not lie in enhancing overall treatment effects but rather allows for an earlier achievement of major therapy goals [23,36], mid-treatment effects were also analyzed. However, no differences between DCS and placebo could be found at mid-treatment. The authors further investigated several moderators, but did not find any of them to be substantial apart from year of publication, with more recent studies showing smaller effects.

Even though the most recent meta-analysis [43] was published not long ago, we believe that the current meta-analysis is important for the following two reasons. First, there are three new high quality studies [40,44,45] that were not included in the analysis of Mataix-Cols et al. [43]. Second, quite a few primary studies used multiple outcomes to assess symptom severity, but meta-analyses appear to have only used one outcome per study. This results in a loss of information and may bias effect sizes due to the selective omission of outcomes. Hence all reported outcomes of each study were included in the present analysis (see the methods section for more details).

Thus, the present paper aims at further investigating whether DCS (vs. placebo) might enhance the effectiveness of cognitive behavioral therapy in anxiety and obsessive-compulsive disorders using more data. Potential moderating variables such as timing and dose of DCS administration, number of treatment sessions, type of disorder, and methodology quality of the primary studies are also investigated.

## Methods and materials

The current meta-analysis was conducted in accordance with the recommendations of the Cochrane Collaboration [46] and the PRISMA guidelines. Inclusion and exclusion criteria, literature search as well as data collection and data analysis are described below. The study was not pre-registered.

### Study selection

The selection of inclusion and exclusion criteria for this meta-analysis was carried out with regard to the criteria applied in the three already published meta-analyses by [15,41,42]. Relating to the primary diagnosis of the study participants, an anxiety disorder or OCD had to be diagnosed on the basis of the Diagnostic and Statistical Manuals of Mental Disorders DSM-IV or DSM-5 [47] or according to the International Statistical Classification of Diseases and Related Health Problems ICD-10 [48]. Nonclinical studies were excluded. Comorbidity with other mental illnesses was permitted as long as they were not the primary diagnosis (based on symptom severity). Alongside to the diagnostic tools DSM and ICD, the symptom severity had to be measured by one or more diagnostic instruments, for instance, self-report inventories or external rating scales. Apart from that, studies were not included if they examined the effects of DCS on other mental disorders than anxiety disorders and obsessive-compulsive disorders (e.g., schizophrenia or autism). Only randomized, double-blinded, placebo-controlled studies were considered to be eligible to ensure the methodological quality of this meta-analysis as these criteria are commonly described as the gold standard of intervention based studies [49]. Further, it was essential that studies examined the effect of DCS on the success of CBT solely, without any influences of other substances. In addition, studies were only included if they focused on CBT with exposure as part of the intervention as a core element for the treatment of anxiety and obsessive-compulsive disorders. Studies that investigated the influence of DCS on other interventions than behavior therapy (e.g., cue exposure, operant conditioning or attention processes) or on the outcome of the treatment of substance-dependence (e.g., alcohol, nicotine, cocaine) were excluded. Another precondition for journal articles to be included was that they reported all data minimally necessary for the statistical analysis that is means, standard deviations, and sample sizes (or equivalent data). If not all relevant information was given, the study authors were contacted and kindly asked to provide the missing data. It was required that data collection had to occur at least twice during the investigation process for both the experimental and control group in order to provide a pre- and post-treatment score of the diagnostic instruments. At last, this meta-analysis only concentrated on DCS augmentation of behavior therapy in humans.

Relevant literature was first identified through a keyword-based search in various electronic databases and performed throughout the period from January 1st, 2016 until January 28th, 2016. In October 2016, the study basis was updated resulting in three new studies [40,44,45]. Journal articles were thoroughly checked in order to determine their value for the present meta-analysis. All of the following working steps including the keyword-based search and the implemented search strategy are described in more detail in the following section. The keyword-based computer search was conducted using the electronic databases *PsychInfo*, *Psyndex*, *PsychArticles*, *PubMed* and *Web of Science* applying the search term

(DCS OR cycloserine OR d-cycloserine) AND (extinction OR exposure therapy OR behavio* therapy OR cognitive therapy OR cognitive behavio* therapy OR CBT)

Literature search was restricted to studies published between October 2007 and November 2016, as all three previous meta-analyses [15,41,42] investigated the literature at least until September 2007. Further, only English and German language studies were considered. No restrictions were formulated for the type of study, peer-reviewed articles were included as well as dissertations or book chapter, mainly for the purpose of avoiding or reducing the risk of a publication bias [50]. 51 [51] discovered that the estimates of treatment success are more likely to be overestimated if authors do not include grey literature in their meta-analyses. In addition, a systematic review by [52] did also find that the results from non-published, grey literature have a significant impact on the outcome of a review as they “tend to show an overall greater treatment effect than grey trials” (p. 5). Thus, it was decided to expand the electronic literature search to one more database called *OpenGrey* (http://www.opengrey.eu/). This database allows researchers to provide scientific content including academic literature that was not formally published in electronic databases or conventional books and scientific journals.

**Fig 1:**
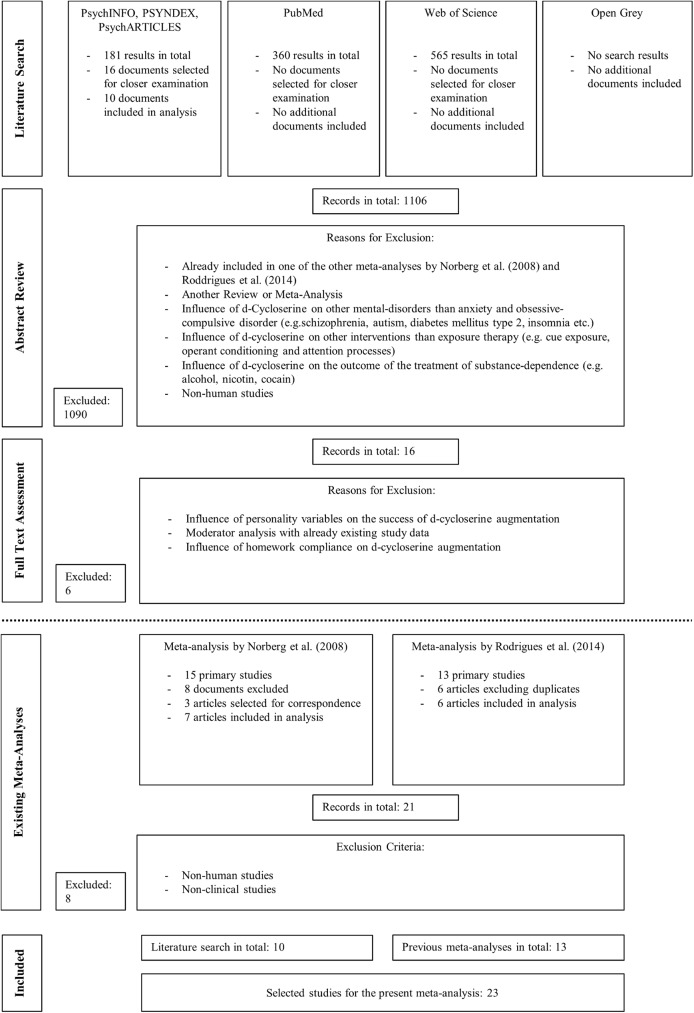
Flowchart illustrating the literature search process.

The search for literature through the above described search process yielded a total of 1106 results. The final study selection was carried out in three hierarchical steps. First, the abstracts of all studies were screened with the purpose of deciding whether the full text of the studies should be reviewed in detail. A large number of documents were excluded based on initial abstract review. In a second step, 16 studies were chosen for closer examination and screened in detail. The final step involved the decision whether or not to include the study in the meta-analysis. Ten studies were considered to be eligible according to the strict inclusion criteria that were previously described. All of these studies were published between 2013 and 2016. No additional study could be found for the period of time which was already covered by the literature search of the previous meta-analyses. Alongside the ten new studies, 13 studies from the previous meta-analyses could be included, resulting in a total of 23 studies for the present meta-analysis. Fig 1 illustrates the search process and the number of articles found and excluded with full details.

### Data collection

In order to compute the effect sizes for this meta-analysis, the mean, standard deviation and sample size for each outcome at pre, mid, post, and follow-up treatment were obtained separately for the experimental and control groups. In some studies the actual results were missing, but graphs or curves providing the required data were available. In order to achieve an accurate and simple extraction of data, the program *g3data 1*.*5*.*3* [53] was applied. As all included studies differed in their sample characteristics, a variety of variables were considered as potential moderators for the subsequent meta-analyses. Each study was coded for their type of sample (adult vs. adolescent), proportion of females, age, and primary diagnosis of the patients, dropout rate as well as relevant comorbidities and concurrent medication. Information on the CBT protocol of individual primary studies was gathered, including the number of CBT sessions in total, the quantity of DCS doses given, dosage of DCS in *mg* and the time of administration before therapy in minutes. Further, validity, test-retest reliability, internal consistency, and inter-rater reliability were coded for the instruments measuring the severity of anxiety and OCD symptoms. Two raters (first and second author) with a degree in psychology coded the studies. Inter-rate agreement was above 98% and disagreements were resolved by consensus.

During the process of conducting a meta-analysis it is of importance to appraise the methodological quality of included primary studies [54]. There is substantial scientific evidence, that studies of poor quality tend to report larger estimates of treatment effects than studies with better methodological quality [55,56], despite existing evidence of the contrary [57]. For the present meta-analysis, a self-developed quality assessment scale was established in accordance with the method proposed by the Cochrane Collaboration [46]. This pursued the goal of examining whether the methodological quality of the included studies has a moderating influence on the study outcome in the current meta-analysis. All studies were rated on an 10-item scale according to the quality scoring system by 58 [58] as *low risk of bias* (coded as 1), *high risk of bias* (coded as 0) and *uncertain risk of bias due to insufficient information* (coded as 0.5). Methodological quality of the included studies is detailed in S1A1 File.

### Statistical analysis

To estimate the effect of DCS on the effectiveness on behavior therapy, we used two types of effect sizes. First, Hedges’ *g* was computed for all time points separately to facilitate comparison with the previous meta-analyses, which also reported Hedges’ *g* estimates. Second, the standardized mean change score (SMCC; 59) for mid, post, and follow-up time points was computed. The SMCC, unlike Hedges’ *g*, not only contrasts treatment and control group, but also controls for possible differences in the pre-treatment values. This is achieved by (1) computing the standardized mean difference between two time points (e.g., pre- and post-treatment) separately for treatment and control group and (2) contrasting the two groups with respect to this difference (see S1B File for the computational details). The SMCC provides a better estimate of the treatment effect, but it requires knowledge of the correlations of outcomes across time points [59]. As these correlations were rarely if ever reported in the primary studies, they were set to *r* = 0.5 in order to be similar to correlations obtained in behavior therapy studies, whose raw data were available to the authors of the present meta-analysis.

Since studies differ more or less in their study design, participants, outcome assessment and treatment properties, certain diversity is likely to appear which can lead to different study outcomes. This diversity is also described as heterogeneity [60]. Statistical heterogeneity in particular occurs when the true effects of the different studies show a larger variation than it would be expected due to random error or by chance [60,61]. With regard to the interpretation of the results and the conclusions that can be drawn from a meta-analysis, it is important to assess the heterogeneity between the studies [62]. Therefore, multilevel (“random effects”) meta-analyses were performed not only allowing us to estimate the pooled effect sizes and corresponding credible (i.e., Bayesian confidence) intervals, but also the between-study variance **τ**^2^ and standard deviation **τ** (see S1C File for details of the applied meta-analytic model).

In some of the primary studies, multiple outcomes were reported. In order to make use of all available information and to avoid potential bias by selecting only one outcome per study, all reported outcomes of each study were included in the meta-analysis. Assuming different outcomes of the same study to be independent is likely invalid as they refer to the same treatment and control group. Thus, outcomes reported by the same study were explicitly modeled as correlated. In the absence of any reported correlations in the primary studies, correlations were set to *r* = 0.7. To investigate the influence of this decision on the obtained results, a sensitivity analysis was conducted where correlations were varied between *r* = 0.1 and *r* = 0.9.

To analyze the influence of potential moderators, meta-regression models [63] were fitted separately for each moderator. Potential for publication bias was examined using funnel plots [64,65] and the trim and fill method [66]. The α-level of all statistical tests was set to α = .05. Whenever hypotheses regarding the effects’ direction could be stated a-priori, one-tailed tests were applied. All computation was done in R (R Core Team, 2015). The package *metafor* [67] was used for the effect size computation, while the package *brms *[68]–allowing to fit Bayesian multilevel models (including meta-analytic models) using *Stan* [69]–was used for the actual analysis.

## Results

### Study characteristics

The current meta-analysis was conducted on the basis of 23 studies which met the inclusion criteria. The complete data as well as R code allowing to reproduce the results is hosted on OSF (https://osf.io/6h87a/). With regard to the overall number of participants included in the primary studies, 659 participants in the experimental group who were given DCS and 655 in the control group who received placebo, combine for a total count of 1314 participants. The mean age of participants included in the studies was 29.2 years, with female attendants accounting for 49% of the sample. With regard to the diagnosis, two studies tested the effects of DCS on behavior therapy on patients with acrophobia including 56 participants [21,38], one study on snake phobia / specific phobia with 20 participants [39], three studies described the treatment of patients with agoraphobia / panic disorder with a total of 246 participants [22,23,40], eight studies including 423 patients with OCD [20,24–29,44], four studies conducted their study on 256 patients suffering from post-traumatic stress disorder [30–33], four studies on social anxiety disorder with a total of 262 included participants [34–37] and finally one more study on mixed anxiety disorders with 51 participants [45]. Quality scores ranged from 6.5 to 10 points with a mean of 8.4. S1A2 File provides an overview of the primary studies included in the present meta-analysis displaying descriptive statistics for pre- and post-treatment as well as demographical and clinical characteristics, including the given diagnoses and instruments used for symptom severity measurement.

### Meta-analysis

**Fig 2:**
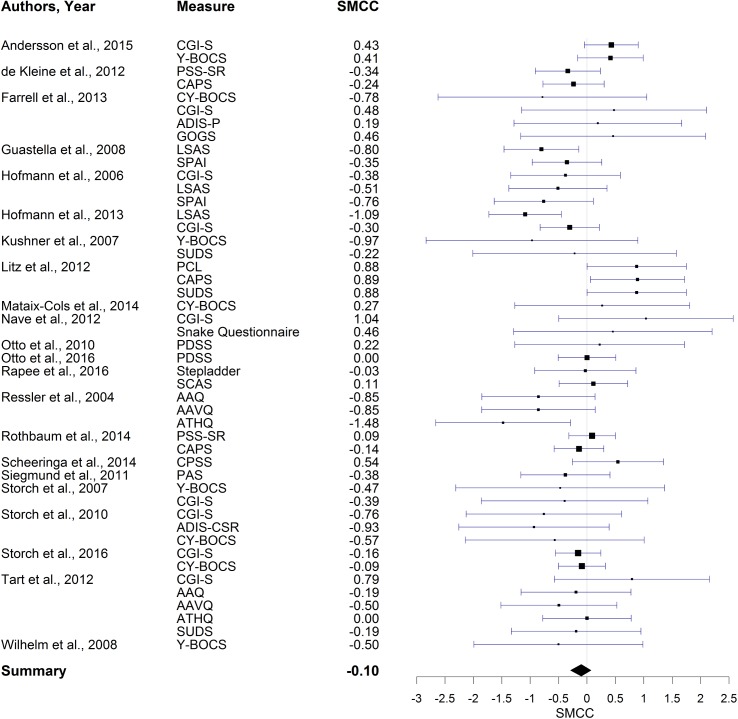
Forest plot of SMCC estimates at post-treatment.

When considering the Hedges’ *g* estimates not controlling for pre-treatment values, DCS appears to have no significant effect on behavior therapy for mid-treatment (*g* = -0.09, *CI* = [-0.28, 0.07], *p* = .14), but a small effect for post-treatment (*g* = -0.12, *CI* = [-0.27, 0.02], *p* < .05) and 1-month follow-up (*g* = -0.27, *CI* = [-0.47, -0.08], *p* < .01). When investigating the SMCC estimates, which take into account possible differences in pre-treatment values, the effect of DCS is not significantly different from zero for mid-treatment (*SMCC* = -0.05, *CI* = [-0.26, 0.15], *p* = .31), post-treatment (*SMCC* = -0.10, *CI* = [-0.29, 0.07], *p* = .13), or 1-month follow-up (*SMCC* = -0.18, *CI* = [-0.46, 0.10], *p* = .10). The main results of the meta-analyses are summarized in Table 1. A forest plot visualizing the obtained SMCC effects of each study at post-treatment can be found in Fig 2 (see S1D File for additional forest plots).

**Table 1:** Main results of the meta-analysis.

Time of Measurement	# Studies	# Outcomes	Effect Size	Estimate	*95%-CI*	*p*-value	τ	*95%-CI* of τ
Pre	23	48	Hedges’ *g*	0.03	[-0.08, 0.14]	.700	0.08	[0.00, 0.20]
Mid	9	17	Hedges’ *g*	-0.09	[-0.28, 0.07]	.138	0.09	[0.00, 0.27]
			SMCC	-0.05	[-0.26, 0.15]	.308	0.09	[0.00, 0.27]
Post	22	47	Hedges’ *g*	-0.12	[-0.27, 0.02]	.044*	0.20	[0.04, 0.35]
			SMCC	-0.10	[-0.29, 0.07]	.132	0.18	[0.01, 0.40]
1-month follow-up	9	20	Hedges’ *g*	-0.27	[-0.47, -0.08]	.002**	0.09	[0.00, 0.26]
			SMCC	-0.18	[-0.46, 0.10]	.104	0.12	[0.00, 0.37]
3-month follow-up	10	21	Hedges’ *g*	0.06	[-0.08, 0.21]	.800	0.07	[0.00, 0.21]
			SMCC	0.14	[-0.07, 0.35]	.904	0.08	[0.00, 0.24]
6-month follow-up	6	10	Hedges’ *g*	0.13	[-0.06, 0.32]	.919	0.08	[0.00, 0.24]
			SMCC	0.23	[-0.05, 0.52]	.948	0.12	[0.00, 0.37]

*Note*: SMCC = standardized mean change score, CI = credible interval, τ = between-study standard deviation. Listed *p*-values are one-tailed as a beneficial effect of DCS was expected a-priori.

* = significant with α = .05

** = significant with α = .01.

### Moderator analysis

We present moderator analysis only for post-treatment, because (a) only very few studies (less or equal to 10) reported effects for the other time points and (b) we did not want to broaden the analysis too much (for sake of completeness, we did compute meta-analysis for mid and follow-up treatment times, but not surprisingly due to the small number of studies, none of the moderators turned out to be significant in these cases). A summary of the effects of all investigated moderators is provided in Table 2 for the SMCC and Table D1 in S1D File for Hedges’ *g*. The vast majority of moderators could not explain any heterogeneity among studies. In particular, no moderator related to the CBT protocol (e.g., number of CBT sessions, dosage of DCS in *mg*, number of DCS doses) had a significant effect.

**Table 2:** Moderator analysis of SMCC estimates at post-treatment.

Moderator	Level	Estimate	*95%-CI*	*p*-value
Time of DCS administration (h)		0.25	[-0.07, 0.56]	.123
Quantity of DCS doses (mg)		0.00	[-0.01, 0.01]	.554
Number of DCS doses		-0.01	[-0.10, 0.08]	.850
Number of CBT sessions		0.02	[-0.08, 0.04]	.496
Length of CBT sessions (min)		0.00	[-0.01, 0.01]	.842
Homework (yes vs. no)	Yes	-0.18	[-0.56, 0.20]	.345
Diagnosis	Specific Phobia	-0.16	[-0.67, 0.35]	.540
	OCD	0.16	[-0.19, 0.48]	.345
	Social Anxiety	-0.37	[-0.76, 0.00]	.050
	PTSD	0.30	[-0.04, 0.66]	.083
	Agoraphobia / Panic	0.08	[-0.38 0.52]	.716
Mean age		-0.01	[-0.03, 0.01]	.274
% females		0.17	[-0.83, 1.23]	.738
Sample (adult vs. adolescent)	Adult	-0.16	[-0.57, 0.23]	.437
% participants taking antidepressants		0.58	[-1.14, 2.26]	.479
% participants taking tranquilizer		1.96	[-1.19, 5.31]	.227
% participants with a comorbid mood disorder		-0.26	[-1.34, 0.79]	.619
Year of publication		0.07	[0.02, 0.12]	.003**
Quality score		0.20	[0.01, 0.40]	.034*

*Note*: SMCC = standardized mean change score, CI = credible interval. Moderator *Diagnosis* is sum-coded; all other categorical moderators are dummy-coded. Listed *p*-values are two-tailed.

* = significant with α = .05

** = significant with α = .01.

**Fig 3:**
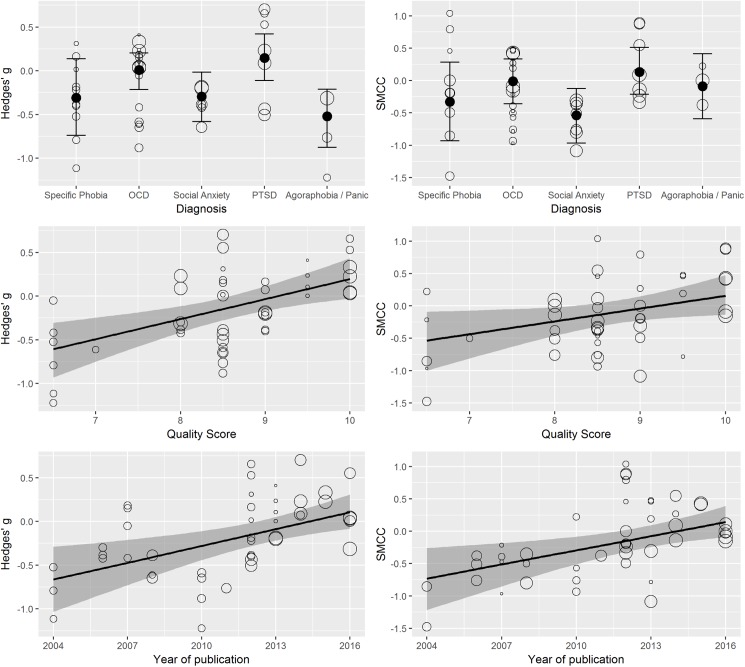
Moderator-analyses of primary diagnosis (top), quality score (middle), and year of publication (bottom). The size of the dots indicates the weights of the observed effect sizes. Error bars indicate 95% credible intervals of the regression line. The specific phobia group contains studies of acrophobia and snake phobia patients.

However, there were three moderators, which appeared to have substantial influence on the observed effect sizes, namely the primary diagnosis of the patients undergoing behavior therapy, the quality score assessing the methodological quality of the studies as well as their year of publication. With regard to the primary diagnosis, studies investigating the effect of DCS on PTSD patients reported higher (i.e., worse) Hedges’ *g* estimates (*b* = 0.34, *CI* = [0.09, 0.60], *p* < .01) than the grand mean of all five investigated diagnosis groups that is specific phobia (i.e., acrophobia and snake phobia), OCD, PTSD, social anxiety, and agoraphobia / panic disorder. The same tendency could be found for the SMCC estimates although it did not quite reach significance (*b* = 0.30, *CI* = [-0.04, 0.66], *p* < .10). Also, studies investigating the effect of DCS on agoraphobia / panic disorder patients reported lower (i.e. better) Hedges’ *g* estimates (*b* = -0.33, *CI* = [-0.64, -0.05], *p* < .05) than the grand mean. However, this could not be found for the SMCC estimates (*b* = 0.08, *CI* = [-0.38, 0.52], *p* = .72). The differences in diagnosis groups are illustrated on the top of Fig 3. From these plots it becomes additionally evident that studies of social anxiety patients reported better effects of DCS than studies of PTSD patients. Indeed, the contrast between these two groups turned out to be significant for Hedges’ *g* (*b* = 0.44, *CI* = [0.06, 0.84], *p* < .05) as well as for the SMCC (*b* = 0.68, *CI* = [0.14, 1.25], *p* < .05).

With regard to the quality score, studies of lower quality tended to report stronger effects of DCS than studies of high quality both for Hedges’ *g* (*b* = 0.23, *CI* = [0.10, 0.37], *p* < .001) and for the SMCC estimates (*b* = 0.20, *CI* = [0.01, 0.40], *p* < .05). This finding is in line with previous studies reporting greater effects in studies with lower methodological quality [55,56]. The relation of the quality score with the observed effect sizes is illustrated in the middle of Fig 3. Year of publication was also positively related to the DCS effect (*b* = 0.06, *CI* = [0.02, 0.11], *p* < .01 for Hedges’ *g*; *b* = 0.07, *CI* = [0.02, 0.12], p < .01 for the SMCC) implying that more recent studies reported smaller effects as illustrated at the bottom of Fig 3. Note that study quality and year of publication are highly confounded having a correlation of *r* = 0.59, which shows that more recent studies are of higher methodological quality. Thus, the effects of these two moderators should not be interpreted independently. In the meta-analysis of Mataix-Cols et al. [43], year of publication was the statistically stronger moderator, but we believe that one should think of it rather as a proxy of study quality.

To make sure that the differences between diagnosis groups cannot just be explained by differences in the study quality or year of publication, we also computed moderator-analyses including all three moderators at the same time, which did not lead any substantial change in the results.

### Sensitivity analysis

In order to investigate the robustness of the above discussed effects, several additional analyses were performed. Most importantly, potential for publication bias was investigated. According to the funnel plots of the post-treatment values displayed Fig E1 in S1E File, there seems to be little evidence of publication bias. Results obtained by the trim an fill method [66] confirm this finding (one study missing on the right-hand side of the funnel for Hedges’ *g* estimates; no study missing for the SMCC estimates). Funnel plots and the trim and fill method were also applied for the other time points. Some evidence of publication bias was found at 1-month follow-up, as multiple studies with small effect sizes appeared to be missing on the right hand side of the funnel (see S1E File).

Conducting a meta-analysis comes with many researchers degrees of freedom [70]. With respect to the statistical analysis, one main decision that had to be made was the value of the correlation between effect sizes of the same sample. In the above analyses, it was set to *r* = 0.7. To investigate the sensitivity of the results on this correlation, we varied its values between *r* = 0.1 and *r* = 0.9. Overall, results changed only very little with varying correlation (see Table D2 in S1D File for the results at post-treatment).

## Discussion

The present meta-analysis investigated whether DCS can augment the effect of behavior therapy in humans with anxiety and obsessive-compulsive disorders. Results suggest that, when aggregating over all studies, the post-treatment effect of DCS is very small and almost indistinguishable from zero when controlling for pre-treatment values (*g* = -0.12; SMCC = -0.10). This stands in contrast to previous meta-analyses [15,41–43], which found small to moderate enhancing effects. However, they were based on fewer studies (counting only those investigating the effect of DCS in humans) and did not include multiple effect sizes per study. Accordingly, the present meta-analysis may mark a turning point in the research of DCS suggesting that–overall–it is less promising in enhancing the effect of behavior therapy than initially thought.

Some of the first studies on DCS augmentation by [20,29] observed that participants who received DCS showed a clinically significant faster treatment response. This finding could be replicated in later studies (Storch et al., 2010; Siegmund et al., 2011; de Kleine et al., 2012), although they failed to find an overall effect for DCS augmentation at post-treatment. After getting similar results, Hofmann et al. [36] summarized that “d-cycloserine does not amplify the effects of cognitive behavioral therapy at major endpoints but may temporarily accelerate therapy gains” (p. 755). Investigating this hypothesis in detail is of great importance as a shortened duration of treatment and faster symptom reduction through DCS administration could considerably reduce therapy costs and dropout rates [25,42]. According to this idea, we would expect a greater decrease in symptom severity in the DCS (relative to placebo) group at the beginning of treatment, whereas the placebo group catches up later on. Therefore, it is assumed that the effect of DCS at mid-treatment should be larger than at post-treatment. It should be noted that the pattern found by [36], as shown in their Fig 1, was rather different (the linear slope was just a bit larger in the DCS group) and in our opinion does not really justify their summary quoted above. Results indicated no substantial effect of DCS at mid-treatment (*g* = -0.09; SMCC = -0.05), which is in line with the findings of Mataix-Cols et al. [43]. Thus, the present findings question the idea that DCS temporarily accelerates the effect of behavior therapy for anxiety and obsessive-compulsive disorders.

Taken together, it is questionable whether DCS should be applied at all to augment behavior therapy, because the effects appear to be very small and almost indistinguishable from zero. As we obtained substantial heterogeneity between studies at post-treatment (τ = 0.20 for Hedges’s *g*; τ = 0.18 for the SMCC), several moderator analyses were conducted to identify possible factors that influence the efficacy of DCS. Specifically, we found that the benefit of DCS varies between patient groups: For specific phobia, OCD, and PTSD patients, DCS could not improve the efficacy of behavior therapy at post-treatment, whereas it may have a small to moderate effect for socially anxious patients. When not controlling for pre-treatment values, studies of agoraphobia / panic disorder patients also showed a small improvement through DCS. However, this effect was no longer visible after controlling for pre-treatment values. The difference between clinical groups comes with possible confounders such as the research group undertaking the studies as well as variations in the treatment protocol. Also, we are not aware of any theoretical explanation why DCS augmentation should work in particular for socially anxious patients. Accordingly, these results have to be interpreted with cautions, and more research is needed to investigate and explain possible differences between the clinical groups.

We could also show that the study quality was related to the obtained effect sizes in the way that studies of lower quality found larger effects than studies of higher quality, which is in line with previous findings [55,56]. Despite being worrisome for obvious reasons, it may be additionally problematic because the common publication practice over the last decades was to favor significant / surprising effects over non-significant / non-surprising ones [70], which may have in turn “rewarded” lower quality research. As no evidence of publication bias could be found at post-treatment, it appears that–at least for the present research context–reporting small or null effects did not lead to a measurable disadvantage in the publication process.

Similar to the effect of study quality, year of publication also turned out to be related to the DCS effect with more recent studies reporting substantially smaller effect sizes. This may explain differences in the results between the present meta-analysis and previous ones: While Norberg et al. [15] obtained a moderate to large effect size for human clinical samples (*g* = -0.60), Bontempo et al. [41] reported a moderate effect (*g* = -0.46), Rodriques et al. [42] a small to moderate effect (*g* = -0.34), ending with Mataix-Cols et al. [43] who found only a small effect (*g* = -0.25). Taking into account that the effect size of the current meta-analysis is even smaller (*g* = -0.12), this raises again the question whether the assumption that DCS enhances the effect of behavior therapy for anxiety and obsessive-compulsive disorders is still sustainable in the light of the most recent empirical evidence on this topic.

Because of the fact that all primary studies included in the present meta-analysis differed with regard to their treatment protocol, this offered the opportunity to further investigate whether different variables, for example timing of DCS administration as well as dosage and frequency, have a moderating influence on the effect size obtained. Despite comprehensive research in the last years, no agreement has been reached yet regarding the optimal administration schedule of DCS which is most suitable for enhancing extinction learning in humans suffering from anxiety disorders or OCD [36,42]. In the present meta-analysis none of the moderators related to the treatment protocol could explain a significant amount of heterogeneity between primary studies. This could be due to the fact that many different factors are involved which makes it difficult to attribute effects to isolated parameters without systematic experimental variation. Another possible explanation being driven by the main findings of this meta-analysis it that the effect of DCS is so small that it might not really matter when, how much, or how often DCS is administered.

### Limitations

With regard to the included primary studies, it should be noted that some mental disorders have already been very well investigated and are therefore properly represented in the current meta-analysis. This is the case for OCD, PTSD and social anxiety disorder providing 16 of the included primary studies in sum. In contrast, some disorders such as specific phobia are not adequately represented because of too few studies and comparatively small samples (76 patients). In addition, the systematic literature search on DCS augmentation yielded not a single primary study on generalized anxiety disorder. Given the overall findings of the present meta-analysis it is, however, questionable whether one can expect promising results regarding the DCS augmentation of behavior therapy for these currently underrepresented disorders.

Due to the small number of studies (less or equal to 10) at mid-treatment and follow-up time points, we did not present moderator-analyses for these outcomes as the statistical power would have likely been too low. Further, it should be strongly emphasized that the moderator analyses we did perform for post-treatment outcomes do not allow drawing causal conclusions. For instance, differences in the effect of DCS between diagnosis groups may just be caused by confounders of which there are typically quite a few in meta-analysis [63]. Ideally, we would like to control for at least for some of them, but given the still rather small number of primary studies at post-treatment (22), adding too many moderators to the same meta-regression would have led to a substantial loss of power and general instability of the regression coefficients–as in any other regression analysis with few observations. Accordingly, we primarily investigated the moderators separately. Thus, the results obtained by the present moderator analyses should only be taken as a starting point for further investigation in experimental studies.

Another limitation of the present meta-analysis is the general lack of knowledge of correlations between the same outcomes measured at different points in time as well as of correlations between different outcomes computed for the same sample. In the analysis, the former type of correlation was set to *r* = 0.5 in order to be similar to correlations typically obtained by behavior therapy studies. For the latter type, we performed sensitivity analyses varying the correlation between *r* = 0.1 and *r* = 0.9 showing that is has only very minor influence on the obtained results. Nevertheless, it would be preferable if primary studies also reported relevant correlations in addition to means and standard deviations. Another limitation common across most meta-analyses is that inference can only be made on study or sample level. This is usually sufficient to obtain valid overall estimates as well as estimates for moderators varying at study level. However, it is not ideal when investigating moderators varying at person level [63] such as gender or age. In these cases, the study level summaries (e.g., percentage of females or mean age) are far less informative than the actual raw data, so that estimates based on these summaries only have limited validity. In the light of the recent discussion about open science, it should be stressed that meta-analysis as a whole could be further improved if scientists made their raw data available (if ethically and contractually possible). This would allow us to perform much more detailed analyses, ultimately leading to a better understanding of the underlying mechanisms and influential factors. With respect to the research of DCS in humans, Mataix-Cols et al. [43] performed such a raw data meta-analysis, but the effort to obtain all the (non-publically available) data must have been immense and may not be feasible anymore when the number of primary studies exceeds two or three dozens.

### Conclusion

The present meta-analysis investigated whether DCS can augment the effect of behavior therapy in humans with anxiety and obsessive-compulsive disorders. While previous meta-analysis found small to moderate improvements, the present results including the most recent research indicate that the overall effect of DCS is very small and almost indistinguishable from zero. Only for patients with social anxiety disorder it may be somewhat effective, although it remains unclear whether this can really be attributed to the diagnosis itself. Nonetheless, it should be noted that it is more difficult to detect large treatment effects when comparing two active treatment conditions (behavior therapy with and without DCS), relative to other control conditions such as wait-list control groups. Given that anxiety disorders and OCD are often associated with significant impairment in the person’s daily life, additional augmentation strategies and innovative treatment concepts are needed, particularly for the subgroup of patients that do not seem to fully benefit from the standard intervention strategies. Further, we were able to show that study quality and year of publication were related to the outcomes of the primary studies, in the way that higher quality / more recent studies reported smaller effects of DCS. In sum, these findings raise the question whether DCS should be applied at all to augment behavior therapy for anxiety and obsessive-compulsive disorders.

## Supporting information

S1 File Appendix. (PDF)
