# Supplementary material for: D-cycloserine augmentation of behavior therapy for anxiety and obsessive-compulsive disorders: A meta-analysis
Source: PLoS One. 2017 Mar 10;12(3):e0173660. doi: 10.1371/journal.pone.0173660 (PMC5345832; doi:10.1371/journal.pone.0173660)
Supplement: S1 File — (PDF) [file pone.0173660.s001.pdf]

Appendix A: Study characteristics of the included primary studies

Table A1: Methodological quality of the included primary studies

First Author	Year	Random Sequence Generation	Allocation Concealment	Blinding of Participants and Personnel	Blinding of Outcome Assessment	Incomplete Outcome Data	Selective Reporting	Description of Comorbidities	Description of Treatment Protocol	Description of Concurrent Medication	Description of Concurrent Therapy	Total Quality Score
Andersson	2015	1	1	1	1	1	1	1	1	1	1	10
De Kleine	2012	1	1	1	1	0.5	1	1	1	1	0	8.5
Farrell	2013	1	1	1	1	1	1	0.5	1	1	1	9.5
Guastella	2008	1	1	1	1	0	1	0.5	1	1	1	8.5
Hofmann	2006	0.5	0.5	1	1	1	1	1	1	1	0	8
Hofmann	2013	1	1	1	1	1	1	0.5	1	0.5	1	9
Kushner	2007	0.5	0.5	1	1	0.5	1	0.5	1	0.5	1	7.5
Litz	2012	1	1	1	1	1	1	1	1	1	1	10
Mataix-Cols	2014	1	1	1	1	1	1	1	1	1	0	9
Nave	2012	1	0.5	1	1	1	1	1	1	1	0	8.5
Otto	2010	0.5	0.5	1	1	0.5	1	0.5	1	0.5	0	6.5
Otto	2016	1	1	1	1	0.5	1	0.5	1	1	0	8
Rapee	2016	1	1	1	1	1	1	0.5	1	0	1	8.5
Ressler	2004	1	1	1	1	0.5	1	0	1	0	0	6.5
Rothbaum	2014	1	1	1	1	1	1	0.5	1	0.5	0	8
Sheeringa	2014	1	1	1	1	1	1	0	1	0.5	1	8.5
Sheerin	2013	1	1	1	1	1	1	0	1	0.5	0	7.5
Siegmund	2011	1	1	1	1	1	1	1	1	0.5	0	8.5
Storch	2007	0.5	0.5	1	1	1	1	0.5	1	1	1	8.5
Storch	2010	1	1	1	1	1	1	0.5	1	1	0	8.5
Storch	2016	1	1	1	1	1	1	1	1	1	1	10
Tart	2012	1	1	1	1	1	1	0	1	1	1	9
Wilhelm	2008	0.5	0.5	1	1	0.5	1	1	1	1	0	7.5

Table A2: Main characteristics of the included primary studies

First Author	Year	Disorder	N EG / CG	% Females	Mean age	Symptom measure	Pre-treatment Mean EG / CG	Pre-treatment SD EG / CG	Post-treatment Mean EG / CG	Post-treatment SD EG / CG
Andersson[a]	2015	OCD	64 / 64	58%	34.8	Y-BOCS	23.36 / 22.75	3.53 / 3.80	13.86 / 11.77	6.50 / 5.95
						CGI-S	4.00 / 3.92	0.71 / 0.72	2.83 / 2.58	1.22 / 0.97
De Kleine	2012	PTSD	33 / 34	81%	38.3	CAPS	61.75 / 73.83	16.77 / 16.79	34.33 / 53.65	37.11 / 38.19
						PSS-SR	26.05 / 28.34	8.21 / 8.34	11.80 / 18.41	14.87 / 15.10
Farrell[a]	2013	OCD	9 / 8	59%	13.1	CY-BOCS	30.00 / 28.88	5.47 / 5.80	13.78 / 13.75	5.30 / 7.70
						ADIS-P	6.89 / 6.38	0.78 / 1.06	2.87 / 2.60	2.60 / 2.20
						GOGS	10.78 / 10.25	2.10 / 1.80	6.11 / 5.00	2.73 / 2.39
						CGI-S	5.67 / 5.38	0.70 / 0.70	3.00 / 2.63	1.64 / 1.30
Guastella	2008	SAD	27 / 23	43%	35.5	SPAI	107.53 / 112.13	19.89 / 25.95	89.52 / 99.30	22.63 / 27.26
						LSAS	78.75 / 77.21	20.58 / 25.18	52.86 / 66.68	20.24 / 21.84
Hofmann	2006	SAD	12 / 15	33%	33.7	SPAI	109.88 / 106.21	22.46 / 26.05	81.74 / 96.21	27.01 / 37.74
						LSAS	46.76 / 46.57	15.10 / 20.63	30.65 / 36.67	16.29 / 21.76
						CGI-S	4.80 / 4.84	0.42 / 1.07	3.33 / 3.80	1.23 / 1.15
Hofmann[a]	2013	SAD	87 / 82	43%	32.5	LSAS	81.34 / 81.98	15.06 / 17.15	39.19 / 42.44	10.32 / 20.68
						CGI-	5.33 / 5.26	0.83 / 0.86	2.68 / 2.95	1.41 / 1.43
Kushner	2007	OCD	15 / 17	–	–	Y-BOCS	27.10 / 28.20	3.80 / 5.10	10.90 / 11.20	4.70 / 6.80
						SUDS	82.60 / 85.40	12.80 / 9.40	31.40 / 39.10	18.00 / 17.70
Litz	2012	PTSD	13 / 13	–	32.2	CAPS	69.85 / 73.38	23.24 / 16.35	72.33 / 53.73	28.63 / 26.22
						PCL	37.85 / 39.00	8.76 / 8.77	34.11 / 24.18	21.09 / 14.95
						SUDS	37.85 / 39.00	8.76 / 8.77	34.11 / 24.18	21.09 / 14.95
Mataix-Cols[a]	2014	OCD	13 / 14	49%	14.9	CY-BOCS	26.90 / 25.00	3.70 / 3.40	10.60 / 10.10	7.40 / 6.10
Nave	2012	Snake Phobia	10 / 10	60%	36.8	SQ	22.00 / 23.90	3.43 / 2.24	9.70 / 9.60	6.18 / 5.99
						CGI-S	4.50 / 4.50	0.71 / 0.71	3.00 / 2.70	1.15 / 0.68
Otto	2010	Agoraphobia / Panic	13 / 14	50%	35.0	PDSS	13.47 / 14.14	4.58 / 2.21	3.65 / 6.85	1.87 / 3.03
Otto[a]	2016	Agoraphobia / Panic	88 / 92	59%	35.4	PDSS	13.30 / 13.37	4.50 / 3.39	5.25 / 6.42	3.66 / 3.74

Rapee[a]	2016	Multiple Disorders	27 / 24	49%	9.2	Stepladder	5.65 / 5.60	1.56 / 1.57	2.51 / 2.50	1.77 / 1.76
						SCAS	7.79 / 6.00	3.53 / 3.58	5.58 / 3.42	3.95 / 3.72
Ressler	2004	Acrophobia	17 / 10	59%	45.6	AAQ	73.40 / 65.80	23.10 / 19.61	35.03 / 51.69	23.05 / 14.73
						AAVQ	24.20 / 18.70	10.31 / 8.54	8.48 / 12.56	8.20 / 6.29
						ATHQ	53.90 / 54.40	7.01 / 5.38	27.18 / 42.12	12.29 / 14.17
Rothbaum[a]	2014	PTSD	53 / 53	6%	34.6	CAPS	85.30 / 82.60	22.86 / 24.67	65.90 / 63.80	20.68 / 25.76
						PSS-SR	32.90 / 32.40	11.79 / 11.97	27.10 / 24.20	10.16 / 14.33
Sheeringav[a]	2014	PTSD	29 / 28	56%	12.5	CPSS	33.30 / 26.30	10.90 / 9.20	16.80 / 9.30	12.50 / 8.00
Sheerin[a]	2013	SAD	7 / 9	63%	19.8	SPAI	91.14 / 90.33	22.09 / 19.05	–	–
Siegmund	2011	Agoraphobia / Panic	20 / 19	46%	37.6	PAS	23.23 / 26.13	10.04 / 10.24	11.95 / 17.61	5.81 / 8.50
Storch	2007	OCD	12 / 12	50%	29.0	Y-BOCS	30.10 / 30.60	3.80 / 4.90	10.10 / 8.60	6.80 / 8.80
						CGI-S	4.30 / 4.30	0.80 / 1.10	1.80 / 1.60	1.10 / 1.40
Storch	2010	OCD	15 / 15	37%	12.2	CY-BOCS	24.10 / 26.00	4.40 / 3.80	6.80 / 11.00	6.00 / 6.60
						CGI-S	4.60 / 5.10	0.83 / 0.74	2.00 / 3.00	1.00 / 1.20
						ADIS-P	5.10 / 5.30	0.79 / 0.70	1.50 / 2.50	1.50 / 1.80
Storch[a]	2016	OCD	70 / 72	54%	13.9	CY-BOCS	24.33 / 22.93	7.90 / 7.88	13.98 / 13.53	10.16 / 10.00
						CGI-S	3.67 / 3.43	1.11 / 1.13	2.28 / 2.23	1.45 / 1.47
Tart	2012	Acrophobia	15 / 14	–	33.4	AAQ	48.27 / 58.36	20.98 / 25.71	25.47 / 33.08	14.83 / 22.54
						AAVQ	20.73 / 23.06	7.74 / 10.60	9.00 / 12.38	7.41 / 9.72
						ATHQ	45.67 / 47.71	8.53 / 12.98	45.67 / 47.71	8.53 / 12.98
						SUDS	68.07 / 71.29	14.78 / 19.94	29.73 / 35.55	25.67 / 25.18
						CGI-S	4.13 / 4.14	0.52 / 0.36	2.53 / 2.36	1.13 / 0.81
Wilhelm	2008	OCD	10 / 13	–	39.1	Y-BOCS	26.50 / 25.50	5.00 / 4.20	10.20 / 14.50	7.20 / 6.40

Note: – indicates not available data. [a] Study not included in previous meta-analyses. Disorders: OCD = obsessive-compulsive disorder, PTSD = post-traumatic stress disorder, SAD = social anxiety disorder. Questionnaires: AAQ = Acceptance and Action Questionnaire, AAVQ = Acrophobia Questionnaire with Avoidance, ATHI = Attitudes Toward Heights Inventory, SUDS = Subjective Units of Distress Scale, CGI-S = Clinical Global Impression Scale, PDSS = Panic Disorder Severity Scale, PAS = Panic and Agoraphobia Scale, CY-BOCS = Children's Yale-Brown Obsessive Compulsive Scale, ADIS-P = Anxiety Disorders Interview Schedule for Parents, GOGS = National Institute of Mental Health Global Obsessive-Compulsive Scale, Y-BOCS = Yale-Brown Obsessive Compulsive Scale, ADIS = Anxiety Disorders Interview Schedule, CAPS = Clinician-Administered PTSD Scale, PSS-SR = PTSD Symptom Scale-Self-Report Version, PCL-M = PTSD Checklist-Military Version, CPSS = Child PTSD Symptom Scale, SPAI = Social Phobia and Anxiety Inventory, LSAS = Liebowitz Social Anxiety Scale.

Appendix B: Computation of effect sizes

Appendix B1: Computation of Hedges' *g*

$$g = J * \frac{mean_{EG} - mean_{CG}}{sd_{pooled}}$$

The pooled standard deviation is given by

$$sd_{pooled} = \sqrt{\frac{(n_{EG} - 1)\, sd_{EG}^2 + (n_{CG} - 1)\, sd_{CG}^2}{n_{EG} + n_{CG} - 2}}$$

The correction factor to achieve an unbiased estimator is defined as

$$J = 1 - \frac{3}{4(n_{EG} + n_{CG} - 2) - 1}$$

The variance of Hedges' *g* is given by

$$Var(g) = \frac{n_{EG} + n_{CG}}{n_{EG}\, n_{CG}} + \frac{g^2}{2(n_{EG} + n_{CG})}$$

Appendix B2: Computation of the SMCC

$$SMCC = SMCC_{EG} - SMCC_{CG}$$

The SMCC for experimental group (EG) and control group (CG) is each computed as follows:

$$SMCC_G = J * \frac{mean_{G,t1} - mean_{G,t0}}{sd_{diff}}$$

The indices ***t0*** and ***t1*** indicate different measurement times (e.g., pre- and post-treatment).

The standard deviation of the difference is given by

$$sd_{diff} = \sqrt{sd_{G,t1}^2 + sd_{G,t0}^2 - 2r_{t1,t0}\, sd_{G,t1} sd_{G,t0}}$$

The correction factor to achieve an unbiased estimator is defined as

$$J = 1 - \frac{3}{4(n_G - 1) - 1}$$

The variance of the SMCC for each group is

$$Var(SMCC_G) = \frac{1}{n_G} + \frac{{SMCC_G}^2}{2n_G}$$

The variance of the final SMCC estimate is given by

$$Var(SMCC) = Var(SMCC_{EG}) + Var(SMCC_{CG})$$

Appendix C: Formulation of the meta-analytic model

Let ***Y*** be the vector of observed effect sizes (e.g., Hedges' g estimates), ***X*** the fixed effects design matrix (including an intercept column) containing data of potential moderators, $\boldsymbol{\beta}$ the fixed effects coefficients, and ***V*** the covariance matrix of ***Y***. Then, the multivariate normal meta-analytic model applied in the present study is given as follows:

$$\boldsymbol{Y} \sim \boldsymbol{N}(\boldsymbol{X\beta}, \boldsymbol{V})$$

with

$$\boldsymbol{V_{ii}} = \boldsymbol{Var}(\boldsymbol{Y_i}) + \boldsymbol{\tau}^2$$

$$\boldsymbol{V_{ij}} = \boldsymbol{r_{ij}}\sqrt{\boldsymbol{Var}(\boldsymbol{Y_i})\boldsymbol{Var}(\boldsymbol{Y_j})} \quad (\boldsymbol{i} \neq \boldsymbol{j})$$

The sample variances $\boldsymbol{Var}(\boldsymbol{Y_i})$ are assumed be to known (see Appendix B for their computation). The between effect size correlations are set to $\boldsymbol{r_{ij}} = \mathbf{0.7}$ if ***i*** and ***j*** correspond to effect sizes of the same sample (value varied in a sensitivity analysis) and to $\boldsymbol{r_{ij}} = \mathbf{0}$ otherwise.

The fixed effects coefficients $\boldsymbol{\beta}$ have an improper flat prior over the reals. The parameter $\boldsymbol{\tau}$ denoting the random effects standard deviation has a wide half-cauchy prior, which is only weakly informative but ensures that very large values of $\boldsymbol{\tau}$**,** which are implausible for the scale of ***Y,*** receive less prior weight. As these prior distributions are non- or weakly informative, their influence on the obtained results can be considered as negligible. Model fitting is performed using Markov chain Monte Carlo (MCMC) methods, more specifically the No-U-Turn (NUTS) sampler implemented in Stan.

Appendix D: Additional results

Table D1: Moderator analysis of Hedges' *g* estimates at post-treatment

**Moderator**	**Level**	**Estimate**	***95%-CI***	***p*-value**
Time of DCS administration (h)		0.09	[-0.12, 0.30]	.393
Quantity of DCS doses (mg)		0.00	[-0.01, 0.01]	.792
Number of DCS doses		0.03	[-0.03, 0.09]	.355
Number of CBT sessions		0.00	[-0.04, 0.05]	.876
Length of CBT sessions (min)		0.00	[-0.01, 0.01]	.955
Homework (yes vs. no)	Yes	-0.17	[-0.47, 0.13]	.265
Diagnosis	Specific Phobia	0.11	[-0.48, 0.26]	.555
	OCD	0.20	[-0.01, 0.41]	.064
	Social Anxiety	-0.10	[-0.36, 0.16]	.438
	PTSD	0.34	[0.09, 0.60]	.007**
	Agoraphobia / Panic	-0.33	[-0.64, -0.05]	.028*
Mean age		-0.02	[-0.03, 0.00]	.012**
% females		-0.26	[-1.27, 0.74]	.591
Sample (adult vs. adolescent)	Adult	-0.31	[-0.63, -0.01]	.042*
% participants taking antidepressants		0.69	[-0.49, 1.86]	.242
% participants taking tranquilizer		0.04	[-3.42, 3.81]	.990
% participants with a comorbid mood disorder		-0.05	[-0.92, 0.77]	.861
Year of publication		0.06	[0.02, 0.11]	.002**
Quality score		0.23	[0.10, 0.37]	< .001***

Note: CI = credible interval. Moderator *Diagnosis* is sum-coded; all other categorical moderators are dummy-coded. Listed *p*-values are two-tailed. * = significant with α = .05, ** = significant with α = .01, *** = significant with α = .001

Table D2: Sensitivity analysis of meta-analytic findings at post-treatment

**Correlation between effect sizes of the same sample**	**Effect Size**	**Estimate**	***95%-CI***	***p*-value**
0.1	Hedges' *g*	-0.12	[-0.25, -0.00]	.026*
0.3	Hedges' *g*	-0.12	[-0.25, 0.00]	.029*
0.5	Hedges' *g*	-0.12	[-0.25, 0.01]	.036*
0.7	Hedges' *g*	-0.12	[-0.27, 0.02]	.044*
0.9	Hedges' *g*	-0.14	[-0.35, 0.05]	.076
0.1	SMCC	-0.12	[-0.27, 0.03]	.053
0.3	SMCC	-0.11	[-0.26, 0.03]	.060
0.5	SMCC	-0.11	[-0.26, 0.05]	.084
0.7	SMCC	-0.10	[-0.29, 0.07]	.132
0.9	SMCC	-0.10	[-0.40, 0.20]	.247

Note: SMCC = standardized mean change score, *CI* = credible interval. Listed *p*-values are one-tailed.
* = significant with $\alpha = .05$.

Appendix D: Additional forest plots

**Authors, Year**	**Measure**	**Hedges' g**
Andersson et al., 2015	CGI-S	0.23
	Y-BOCS	0.33
de Kleine et al., 2012	PSS-SR	-0.44
	CAPS	-0.51
Farrell et al., 2013	CY-BOCS	0.00
	CGI-S	0.24
	ADIS-P	0.11
	GOGS	0.41
Guastella et al., 2008	LSAS	-0.65
	SPAI	-0.39
Hofmann et al., 2006	CGI-S	-0.38
	LSAS	-0.30
	SPAI	-0.42
Hofmann et al., 2013	LSAS	-0.20
	CGI-S	-0.19
Kushner et al., 2007	Y-BOCS	-0.05
	SUDS	-0.42
Litz et al., 2012	PCL	0.53
	CAPS	0.66
	SUDS	0.53
Mataix-Cols et al., 2014	CY-BOCS	0.07
Nave et al., 2012	CGI-S	0.31
	Snake Questionnaire	0.02
Otto et al., 2010	PDSS	-1.22
Otto et al., 2016	PDSS	-0.31
Rapee et al., 2016	Stepladder	0.01
	SCAS	0.55
Ressler et al., 2004	AAQ	-0.79
	AAVQ	-0.52
	ATHQ	-1.11
Rothbaum et al., 2014	PSS-SR	0.23
	CAPS	0.09
Scheeringa et al., 2014	CPSS	0.70
Siegmund et al., 2011	PAS	-0.77
Storch et al., 2007	Y-BOCS	0.18
	CGI-S	0.15
Storch et al., 2010	CGI-S	-0.88
	ADIS-CSR	-0.59
	CY-BOCS	-0.65
Storch et al., 2016	CGI-S	0.03
	CY-BOCS	0.04
Tart et al., 2012	CGI-S	0.16
	AAQ	-0.40
	AAVQ	-0.39
	ATHQ	-0.19
	SUDS	-0.22
Wilhelm et al., 2008	Y-BOCS	-0.61
**Summary**		**-0.12**

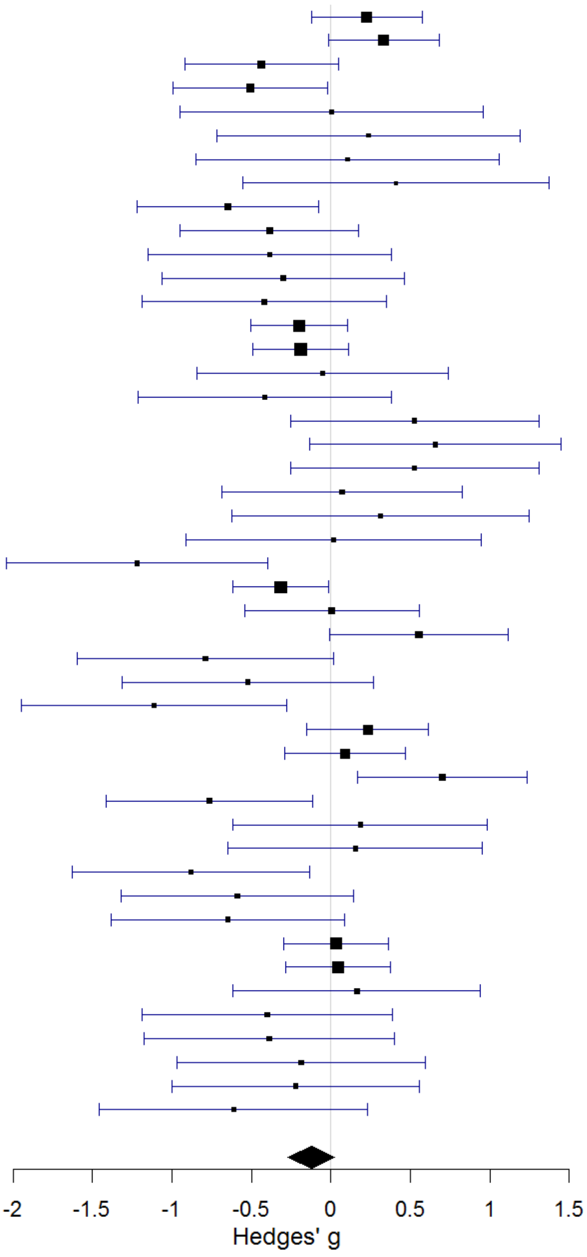

Figure D1: Forest plot of Hedges' *g* estimates at post-treatment.

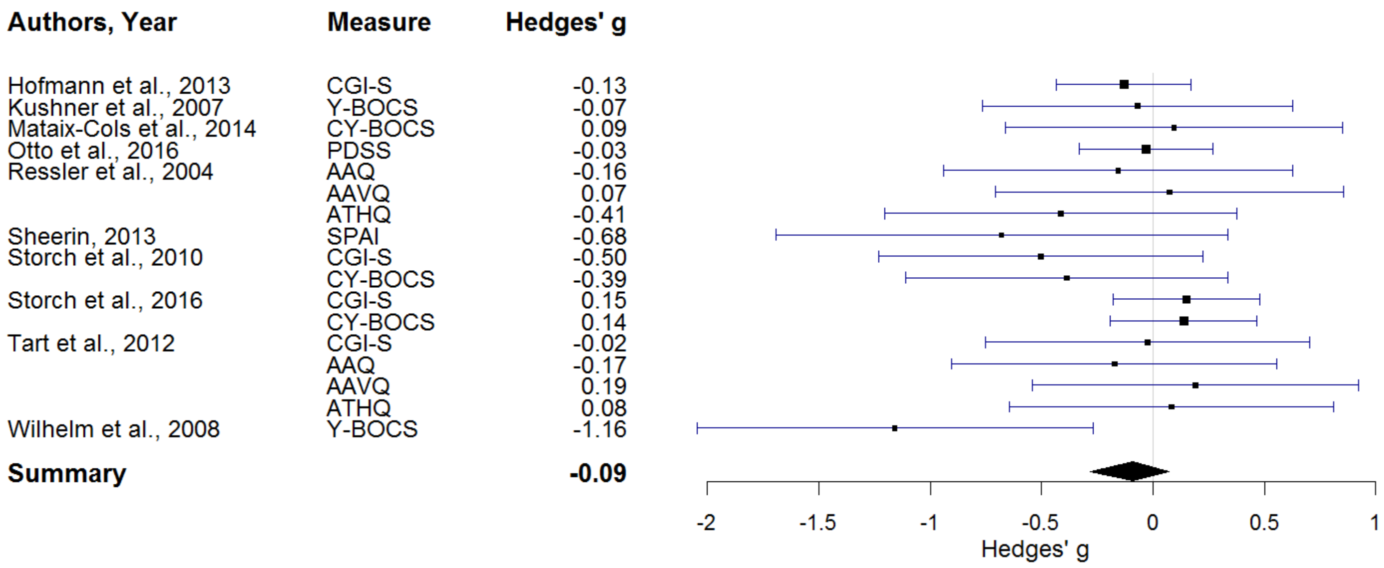

Figure D2: Forest plot of Hedges' *g* estimates at mid-treatment.

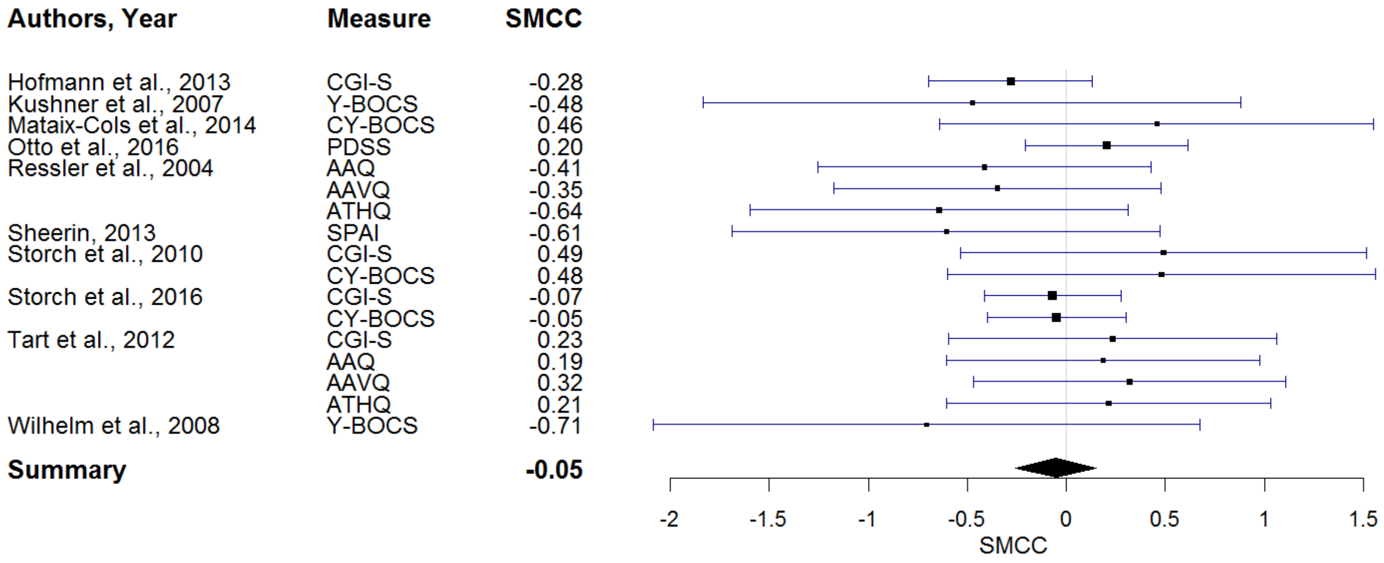

Figure D3: Forest plot of SMCC estimates at mid-treatment.

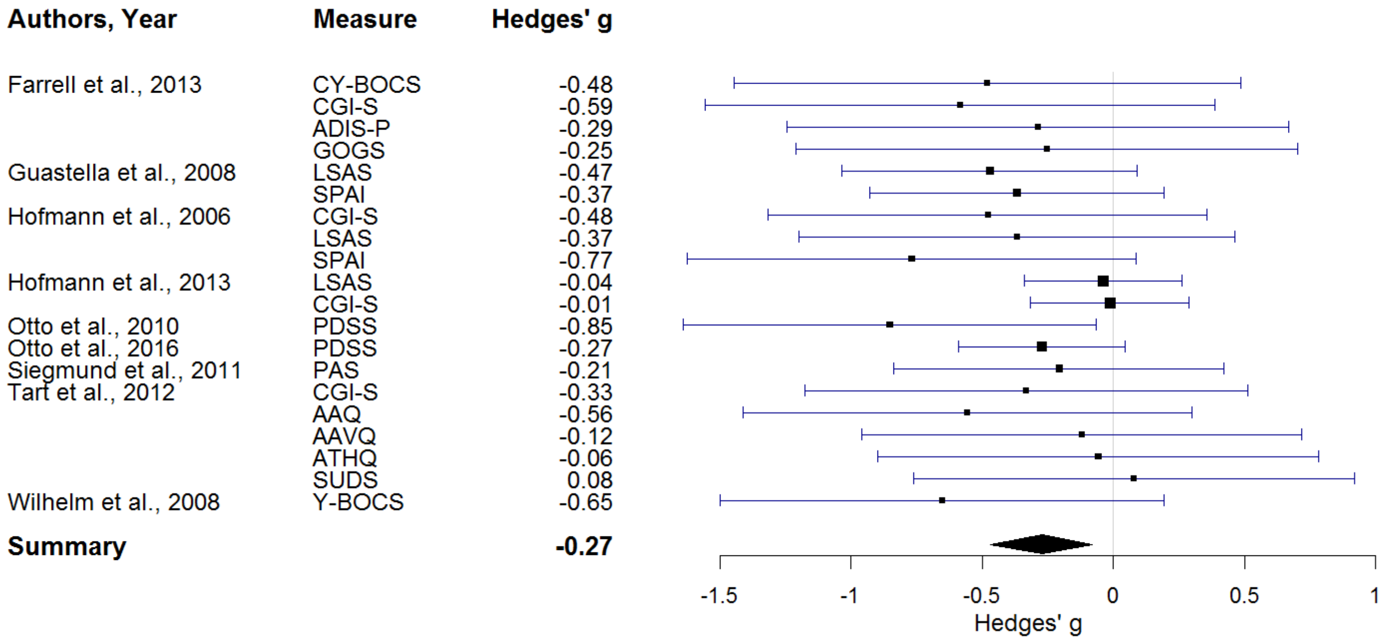

Figure D4: Forest plot of Hedges' *g* estimates at 1-month follow-up.

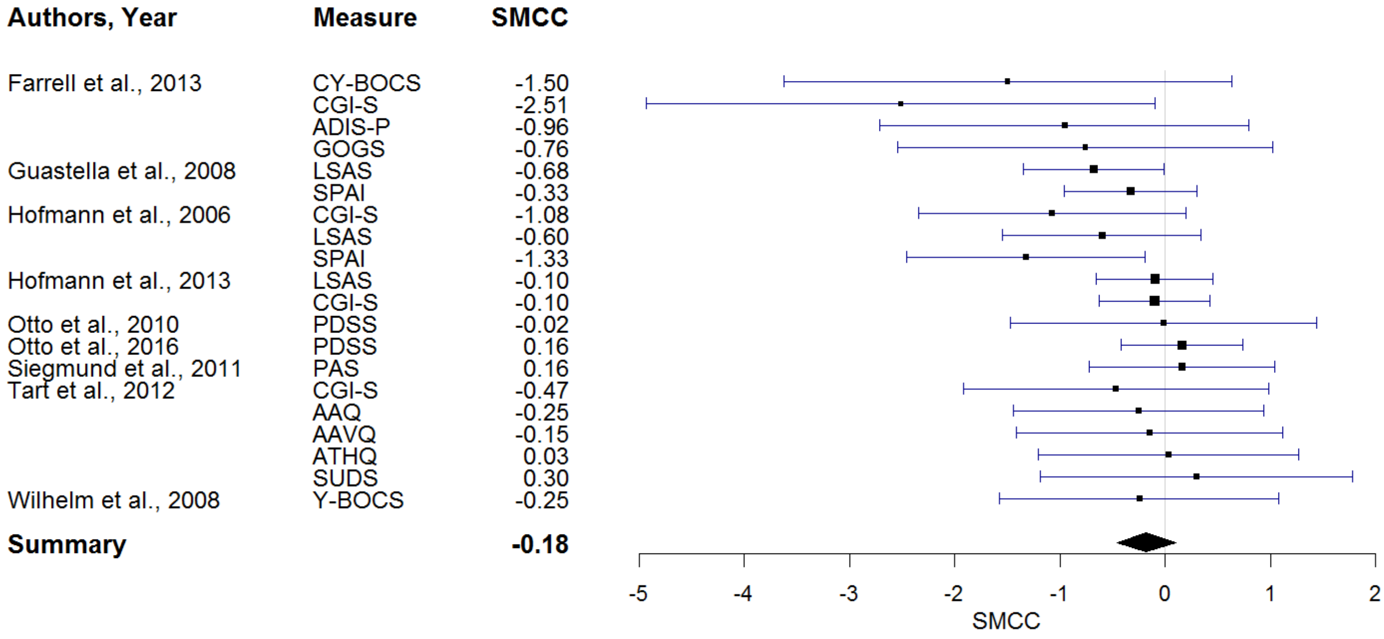

Figure D5: Forest plot of SMCC estimates at 1-month follow-up.

Appendix E: Funnel plots

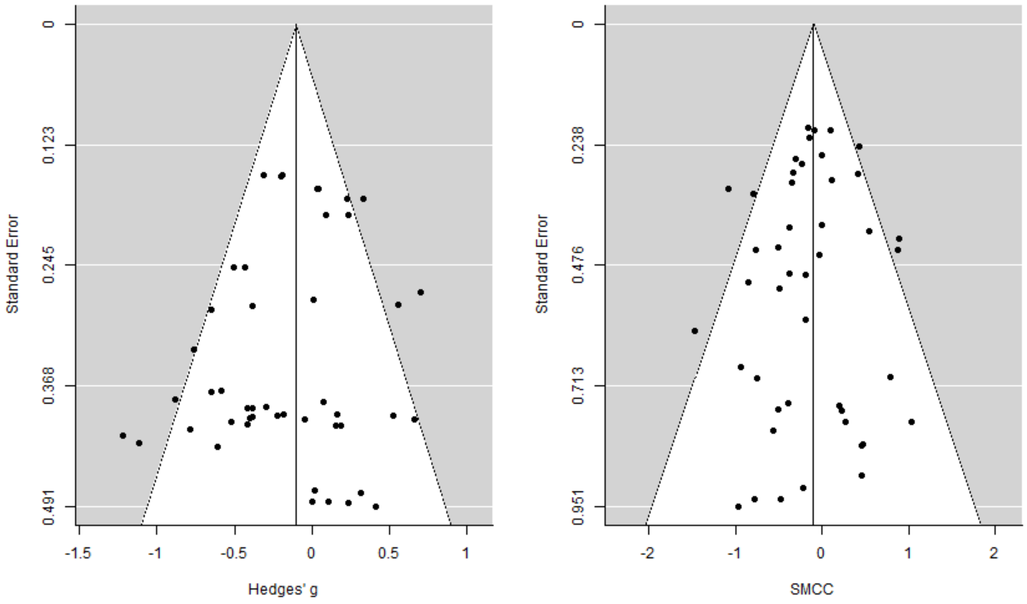

Figure E1: Funnel plots of effect sizes at post-treatment.

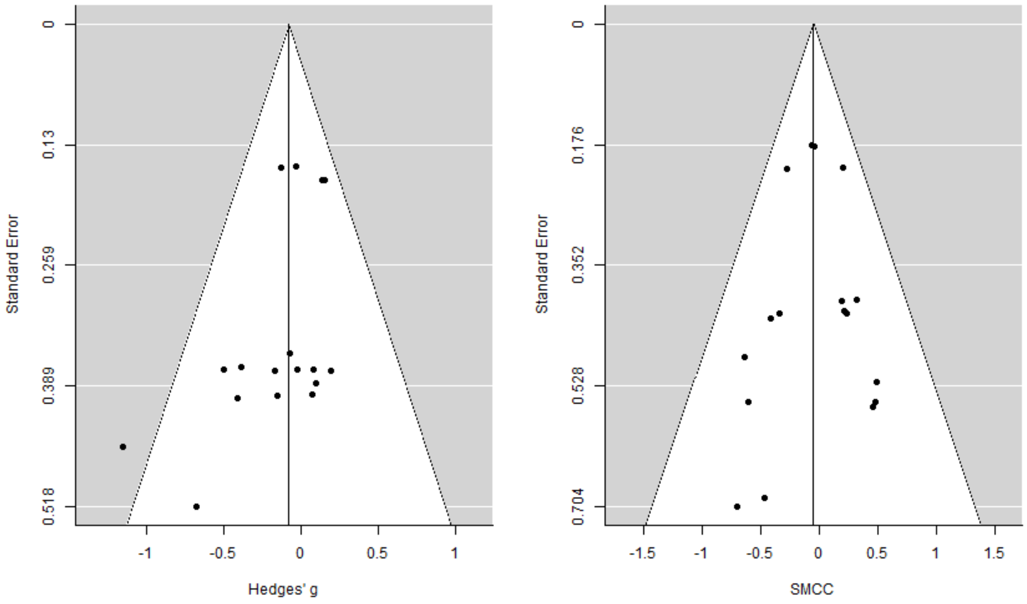

Figure E2: Funnel plots of effect sizes at mid-treatment.

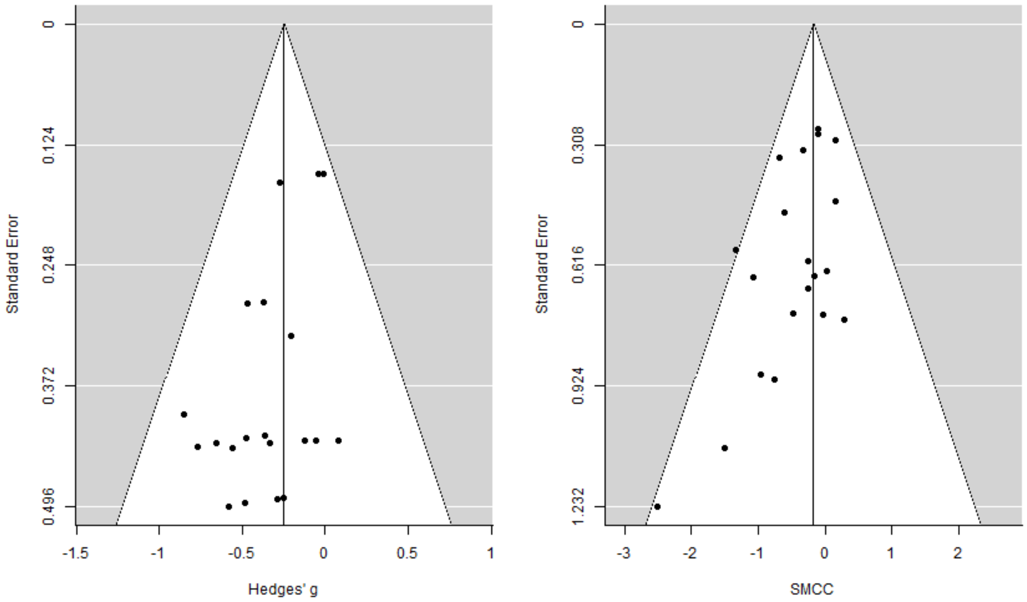

Figure E3: Funnel plots of effect sizes at 1-month follow-up.

PRISMA 2009 Checklist

Section/topic	#	Checklist item	Reported on page #
**TITLE**			
Title	1	Identify the report as a systematic review, meta-analysis, or both.	1
**ABSTRACT**			
Structured summary	2	Provide a structured summary including, as applicable: background; objectives; data sources; study eligibility criteria, participants, and interventions; study appraisal and synthesis methods; results; limitations; conclusions and implications of key findings; systematic review registration number.	2
**INTRODUCTION**			
Rationale	3	Describe the rationale for the review in the context of what is already known.	4-7
Objectives	4	Provide an explicit statement of questions being addressed with reference to participants, interventions, comparisons, outcomes, and study design (PICOS).	6
**METHODS**			
Protocol and registration	5	Indicate if a review protocol exists, if and where it can be accessed (e.g., Web address), and, if available, provide registration information including registration number.	6
Eligibility criteria	6	Specify study characteristics (e.g., PICOS, length of follow-up) and report characteristics (e.g., years considered, language, publication status) used as criteria for eligibility, giving rationale.	7 - 9
Information sources	7	Describe all information sources (e.g., databases with dates of coverage, contact with study authors to identify additional studies) in the search and date last searched.	8 - 9
Search	8	Present full electronic search strategy for at least one database, including any limits used, such that it could be repeated.	9
Study selection	9	State the process for selecting studies (i.e., screening, eligibility, included in systematic review, and, if applicable, included in the meta-analysis).	7 - 9
Data collection process	10	Describe method of data extraction from reports (e.g., piloted forms, independently, in duplicate) and any processes for obtaining and confirming data from investigators.	9 - 10
Data items	11	List and define all variables for which data were sought (e.g., PICOS, funding sources) and any assumptions and simplifications made.	9 - 10
Risk of bias in individual studies	12	Describe methods used for assessing risk of bias of individual studies (including specification of whether this was done at the study or outcome level), and how this information is to be used in any data synthesis.	10
Summary measures	13	State the principal summary measures (e.g., risk ratio, difference in means).	10
Synthesis of results	14	Describe the methods of handling data and combining results of studies, if done, including measures of consistency (e.g., I^2) for each meta-analysis.	11 - 12

PRISMA 2009 Checklist

Section/topic	#	Checklist item	Reported on page #
Risk of bias across studies	15	Specify any assessment of risk of bias that may affect the cumulative evidence (e.g., publication bias, selective reporting within studies).	12
Additional analyses	16	Describe methods of additional analyses (e.g., sensitivity or subgroup analyses, meta-regression), if done, indicating which were pre-specified.	12
**RESULTS**			
Study selection	17	Give numbers of studies screened, assessed for eligibility, and included in the review, with reasons for exclusions at each stage, ideally with a flow diagram.	12 – 13
Study characteristics	18	For each study, present characteristics for which data were extracted (e.g., study size, PICOS, follow-up period) and provide the citations.	12 – 13
Risk of bias within studies	19	Present data on risk of bias of each study and, if available, any outcome level assessment (see item 12).	App. A
Results of individual studies	20	For all outcomes considered (benefits or harms), present, for each study: (a) simple summary data for each intervention group (b) effect estimates and confidence intervals, ideally with a forest plot.	App. A
Synthesis of results	21	Present results of each meta-analysis done, including confidence intervals and measures of consistency.	13 - 15
Risk of bias across studies	22	Present results of any assessment of risk of bias across studies (see Item 15).	16
Additional analysis	23	Give results of additional analyses, if done (e.g., sensitivity or subgroup analyses, meta-regression [see Item 16]).	16
**DISCUSSION**			
Summary of evidence	24	Summarize the main findings including the strength of evidence for each main outcome; consider their relevance to key groups (e.g., healthcare providers, users, and policy makers).	16 - 18
Limitations	25	Discuss limitations at study and outcome level (e.g., risk of bias), and at review-level (e.g., incomplete retrieval of identified research, reporting bias).	20 - 21
Conclusions	26	Provide a general interpretation of the results in the context of other evidence, and implications for future research.	21 - 22
**FUNDING**			
Funding	27	Describe sources of funding for the systematic review and other support (e.g., supply of data); role of funders for the systematic review.	22

From: Moher D, Liberati A, Tetzlaff J, Altman DG, The PRISMA Group (2009). Preferred Reporting Items for Systematic Reviews and Meta-Analyses: The PRISMA Statement. PLoS Med 6(7): e1000097. doi:10.1371/journal.pmed1000097

For more information, visit: **www.prisma-statement.org**.
